# Practical Medicine

**Published:** 1877-09

**Authors:** 


### PRACTICAL MEDICINE.

THE CURABILITY OF INSANITY.—Earle—(*American Jour. of Insanity*, April, '77.) That great injustice is done by superintendents of the institutions for the insane, to the public, by almost universally reporting recoveries of cases rather than of persons, is supported by statistics from twenty institutions for the treatment of the insane; showing a much larger percentage of recoveries than really occurs. The author says that, no patient ought to be reported as recovered twice or more within the same year, and that cases and not persons are constantly being reported, which make the percentage of cases much larger than actually exists. Such unreliable statistical knowledge is chiefly due to a spirit of rivalry among superintendents. There occurred in the Friend's Asylum, eighty-seven admissions, which contributed two hundred and seventy-four recoveries to the statistics of that institution. One patient recovered fifteen times; another, thirteen; a third, nine; a fourth, eight, and a fifth seven times; making fifty-two recoveries; three of these five patients afterwards died in the hospital. Of the State Hospital at Worcester, from its organization, two thousand nine hundred and forty-nine patients were admitted; six hundred and ninety-four were readmissions. Seven persons were admitted an aggregate of one hundred and six times; one was admitted twenty-three times, one eighteen, one sixteen, one fourteen, one thirteen, and two eleven times each. One of the seven persons was discharged cured twenty-two times, one sixteen, one thirteen, two eleven times, one ten and one nine times. The seven persons furnished ninety-two recoveries, and yet two of them died in the hospital, and a third is now an inmate of it, considered hopelessly insane. Some of the chief factors which are instrumental in causing such difference in percentages of recoveries of insane people, says the author, are the special characteristics of the persons reporting them, his temperament and psychological individuality, experts before legal tribunals, often err in judgment upon insane cases. In contrast with the above reports, the State Hospital in Maine furnished statistics in the five years from 1846 to 1850, inclusive, of five hundred and eighty-seven patients, which were admitted during that time; of this number, two hundred and eighty-five, or a proportion of forty-eight and fifty-five hundredths per cent., recovered. The same institution again furnished statistics, from 1871 to 1875, inclusive, of nine hundred and fifty-three admissions, and three hundred and forty-nine, or a recovery of thirty-six and sixty-two hundredths per cent. The reports from these hospitals in Maine, approach more nearly the actual percentage of recoveries, than do the other institutions mentioned. There were admitted at the twenty institutions, in the course of the second five years of their operation, fourteen thousand five hundred and sixteen; the recoveries were in number, six thousand six hundred and eighty-nine; the proportion of recoveries to admissions, forty-six and eight hundredths per cent. The number admitted during the last five years of their existence was, twenty-four thousand three hundred and eighty-three; the recoveries, eight thousand three hundred and fifty-four; and the per cent. of recoveries, thirty-four and twenty-six hundredths; these figures relate to cases and not to persons. The author does not believe that more than ten per cent. of insane people are curable, and to accomplish even this, they must have early and prompt medical assistance. W. F. L.

Nocturnal Terrors in very Young Children.—Jules Simon. —(*Gazette Obstetricale*, 5 May, 1877.) Infants at the breast are dyspeptic much oftener than is supposed, and are then subject, during sleep, to starts, and awakenings in fright, which at a later period are the result of true nocturnal terrors. Recently M. Simon was called in the middle of the night, to a little girl of three years who was the victim of a veritable hallucination. This child, convalescent from whooping cough, had started up out of her sleep uttering violent cries, saying that she saw before her an enormous insect which frightened her, and only regained her senses when spoken to; the light was painful to her, but still she would not remain in the dark. This state lasted the rest of the night, but had entirely disappeared the next day. These nocturnal terrors are not very rare among children, and are caused especially by digestive troubles, though they may be entirely independent of them. Inquiry should always be directed to the digestive functions when such a case occurs. But the possibility of poisoning should also be thought of, for certain poisons manifest their effect by painful hallucinations. Stramonium, in particular, determines these accidents, as Dr. Thors has shown in the case of a child who had swallowed a large number of the berries of this plant. In ordinary cases, calmatives, such as bromide of potassium, or belladonna, and a mild purgative answer all the indications. L. W. C.

Deafness as a Sign of Bright's Disease.—M. Dieulafoy—(*La France Médicale*, No. 16, 1877. M. Dieulafoy reports the following case of deafness, occurring in a patient suffering with Bright's disease: Four years ago, the patient had

œdema of the face, polyuria, dyspnœa, then amblyopia, epistaxis, in a word, the symptoms of Bright's disease. These different symptoms gave but little trouble until four months since, when the dyspnœa became greater, and the œdema suddenly generalized over the entire body; the urine became scanty and high colored, as if the parenchymatous nephritis had become interstitial. There was no cardiac hypertrophy, no *bruit de galop*. Examination of the urine showed a notable proportion of albumen, its density was 1021, and the microscope revealed the presence of hyaline cylinders and numerous deformed red globules.

Besides these symptoms, the patient relates that the malady was ushered in by violent pains in the left side of the face, (trigeminal neuralgia) and by a *complete abolition of hearing* on the same side. She is very explicit on this point; deafness of the left ear, with neuralgia and œdema of the left side of the face, marked the début of the malady. Since then the deafness has never improved.

In another patient presenting similar symptoms, the disease was ushered in by severe neuralgic pains in the left side of the face, the pains still persisting up to the present time, and *deafness of the left ear.* The patient noticed the dullness of hearing at the same time as the diminution of sight, about fifteen days after the œdema of the face. The hearing has never completely been restored.

These two examples of deafness occurring under almost identical circumstances, excited the author's attention, and the question presented itself, whether it was simply a coincidence, or whether the auditory alteration was not consecutive to the albuminous nephritis, and he was not unwilling to admit that the same morbid state might determine troubles in the optic and auditory nerves. In his researches upon this subject, the author has found an analogous example related by Rasenstein, who says it is probable that the accident was connected with an œdema of the auditory nerve.

A fourth example of dullness of the hearing of the right side having preceded the œdema in a patient attacked with interstitial nephritis, is mentioned by the author.

M. Dieulafoy draws no conclusion from these cases, but simply calls attention to deafness occurring in the course of Bright's disease. L. W. C.

A Statistical Retrospect of the Trichina Epidemics in Saxony.—Reinhard.—(*Archiv der Heilkunde*, 1877, No. 3 and 4.) In sixteen years, from 1860 to 1876, there were thirteen trichina epidemics. The number of sick persons was 1,266, of which 19, or 1.58 per cent., died.

But very few were infected through eating raw meat; mostly by eating hard, smoked sausages, and other varieties of sausages; over seven-eighths of all poisoned were infected through eating sausages; very few cases can be traced to the eating of ham. Out of the nineteen deaths, three were infected through eating raw meat; two by hard, smoked sausages; eight by eating common sausages, and two by eating ham.

The epidemics were confined almost entirely to large cities and to the thickly populated districts. The source of the infection was traced, twenty-nine times, to butchers, and only five times to meat raised for home consumption. The num-number infected by one trichinosed hog is very small. It is determined that one medium hog furnishes meat for from 140 to 200 people; out of this number, on the average, not one-fourth are infected, and sometimes not more than twelve.

The tax-list showed that in these sixteen years, over 3,960,500 hogs were slaughtered in Saxony. Out of this number thirty-nine gave rise to epidemics, or one in 180,000 hogs. But observations disprove that trichinosis is so scarce among hogs. Microscopical observations were made in many parts of Saxony.

In Ebenbach, O. L., 2,034 hogs were examined, and one was found to be suffering from trichinosis; in Dresden, Dr. Meissner, veterinary surgeon, examined 3,346 hogs, and found four suffering from trichinosis; or one in 839. The most reliable examinations we have come from Braunschweig. Dr. Uhde examined 757,716 hogs, and found ninety-five infected, or one in 8,000.

Taking the average of tables presented, and we find one hog in 7,706 suffering from trichinosis. This would give for Saxony, with her 6,960,000 slaughtered hogs, about 983 trichinosed hogs. Taking the number of epidemics in consideration, we have four in 100 infected hogs giving rise to such a visitation.

The author says: "Many believe that for every infected hog detected and condemned, so many lives have been saved, or rather, so much suffering prevented. But this is far from the truth, as above statistics have shown."

Neither does the author think that microscopical observations can be carried out in large cities with any degree of accuracy whatever. Several epidemics were caused by hogs that had passed microscopical examination. The examinations having been made by men whose ability to perform them could not be doubted.

TWO CASES OF RATTLESNAKE BITE SUCCESSFULLY TREATED BY INJECTIONS OF CARBONATE OF AMMONIA INTO THE VEINS. Knott. (*Philadelphia Med. and Surg. Reporter, July* 21*st and* 28*th*, 1877.) CASE NO. 1. Was seen fifteen minutes after having received a bite on the hand from a rattlesnake; a large drink of whiskey had just been administered; body bathed in profuse clammy perspiration; no pulse at wrist; respiration abdominal and irregular; pupils completely dilated; inability to swallow. A half drachm of the following solution was immediately injected into the superficial veins of the hand:

℞. Ammoniæ carbonatis, grs. xl; aquæ destillatæ ℥ j.

An almost imperceptable pulse at the wrist appeared, but was immediately lost again. A drachm injection of same solution into the cephalic vein produced almost immediately a pulse at the wrist of about thirty to the minute. The second and third injections followed by still greater improvement, and after the fourth, patient appeared as well as before the reception of the bite. On the following morning patient found in perfect stupor; pupils dilated; pulse feeble and irregular. Twenty-five injections of the ammonia were made in the course of an hour, under which the patient gradually returned to consciousness and sight. Patient was now put upon whisky treatment to test its comparative merit. Large doses were given every fifteen minutes, and continued three hours, when he fell back with stupor, with feeble pulse, dilated pupils and abdominal respiration. Prompt relief was brought about by the injections of ammonia, the remedy being also administered by the mouth. Ordered quinine and beef tea. One more relapse occurred on the following day, which was relieved by four injections, after which the patient was convalescent. Discharged cured on the fourth day.

Case No 2. A colored boy was seen about ten hours after reception of the bite. Whisky had been given during the interval. After three injections of the solution of ammonia into the cephalic vein, the boy arose to his feet, without pain or inconvenience.

The following was ordered:

R. Spts. frumenti ℥ xvi; ammoniæ carb. ʒiiss Ft. Sol. Sig. Tablespoonful every two or three hours in water.

Patient was not seen again, but his recovery was affirmed.

The first patient, a man of intelligence, stated that the poison was felt not exceeding two minutes from the reception of the bite; the first symptoms being nausea and giddiness, with loss of sight. One point particularly noticable in the treatment with ammonia; on making an injection while the patient was under the influnece of the poison, the blood that escaped from the veins, by the needle of the syringe, was of an inky appearance. On the second injection, though made on the opposite side and immediately following the first, the blood presented more the appearance of arterial than veinous, showing plainly the powerful effect exerted by the ammonia in changing the condition of the blood.

Dr. K. is of the opinion, from his experience in these cases, that we have but little to fear from the introduction of air into the veins while employing ammonia, believing this agent prevents any serious effect there-from. But little attention was given to prevent the introduction of air, yet no bad effects were observed. The above cases are the first in which this treatment has been employed in this country. The credit of the introduction of this treatment into the profession, is due to Professor Halford, of Australia.

Intestinal Obstruction.—Dr. Southey—(*British Medical Journal*, May). The case was of a young woman, who had three days previously been attacked on board ship, with severe abdominal pain, complete obstruction of the bowels and stercoraceous vomiting. She had been treated by the ship's captain with calomel and jalap powder, every two hours. It was thought possible that obstruction was due to pelvic hematocele, as the attack occurred during her menstrual period; examination, however, failed to confirm the idea. Over the track of the colon it was hollow and resonant, but over the hypogastrium, hard, indicating an empty colon and loaded small intestines. A long tube was passed into the rectum, but only a little fluid could be injected through it, and that caused great pain. The following plan was then tried, with success. The patient was placed in the genu-pectoral position, a long tube inserted in the rectum, and the anus held closely around it. A siphon bottle of soda water was then connected with the tube, the bottle being placed in hot water to increase the elasticity of the gas contained in it. When the valve of the bottle was opened, the gas and water ascended into the rectum, causing enormous abdominal distention, and severe pain.

Two bottles of the soda water were thus used, when the patient felt "something give way or burst," and a copious action of the bowels followed. No medicine was used except subcutaneous injections of morphia. In two days the patient was well enough to undertake a journey. This plan of treatment which seems preferable to the simple injection of fluid, was recommended two years ago, in one of the French reviews.

On Constipation.—Thomson—(*The Medical Record*, May, 1877). Constipation is due to deficient action of the small, and some portion of the large intestines. Of the small intestines, there are two operative causes: deficient secretion, and want of innervation, or muscular action. Deficient secretion in the small intestines may be due to some disturbance of the liver, and constipation as a result, may date from some severe form of fever in which the liver was involved. In such cases there is not a preponderance of fecal accumulation and impaction, but rather, instead, a sluggish action of the bowels, recourse to medicines being necessary to bring about a movement once in four or five days. The symptoms of deficient action of the small intestines other than constipation, are usually negative; the one which gives the patient the most discomfort, is a dull indefinite headache, located in the posterior part of the head, and is best relieved by such remedies as will promote a free discharge of bile. The tongue is not usually large and flabby, but is reddened along the edges and tip. The secretions of the mouth are commonly viscid. The treatment should not consist of mild cathartics, or purgatives, as the condition of the case would be provoked by them, but what is necessary, is to increase the amount of fluid in the intestines by causing the patient to drink a great deal more water than is his custom. The laxative action of the water may best be insured by the addition of some mild saline; the reason of this is the mixture formed by the union of some saline with water does not readily pass from the intestinal mucous membrane into the general system,—water being retained in the intestinal tube by the saline, excites peristaltic action of the bowels, and so produces an evacuation of its contents. To increase the power of action of the intestines, the author uses small doses of quinine, combined with sulph. magnesia, e. g. sulph. mag. ʒi., sulph. quin. gr. i., mixed and drank in a glass of water every morning. It takes, however, usually from one to two weeks before much effect is noticed.

Deficient innervation of the small intestines, as a rule, accompanies constipation in elderly persons, and also in those whose habits are sedentary. The means to be employed to overcome this form of constipation, are quite at variance with those used in the form of constipation just spoken of. If much water is given this class of patients, weakening of their digestive powers, followed by loss of appetite, and heaviness in the head, will be the result. To increase the innervation of the secretory apparatus, in those who are obliged to remain at their occupations, water is applied externally. A sitz bath, with the water as cold as the patient can bear it, and have good reaction follow, will in very many cases work wonders. Sponging the spine and bowels with cold salt water, made as irritant as can be borne, on rising in the morning, is a very efficient method of using water externally. Some cases are benefited by giving the bowels a local shower-bath.

Of the large intestines, the same causes work the same results. Deficient innervation is by far the most common in this form of constipation. Large fecal accumulations, resulting from want of nerve power in the colon, or rectum, are usually present without the patient's knowledge, and rectal abscess may be the result. The treatment consists in keeping the rectum empty by means of enemata, but great care should be exercised against using the syringe every morning for any considerable length of time, as such a habit is likely to become fixed. After using the syringe to remove accumulations in the bowel, other measures for restoring lost innervation to the organ should be instituted. Strychnia used hypodermically into the sub-mucous tissue, often proves very efficient.

